# Validation of Onco-MPI and Geriatric-8 for predicting 2-year mortality in older Indian patients with breast cancer: a prospective observational study

**DOI:** 10.3332/ecancer.2026.2099

**Published:** 2026-03-19

**Authors:** Shivani Sable, Tabassum Wadasadawala, Myvizhi Kannan, Rajiv Sarin, Rima Pathak, Revathy Krishnamurthy, Seema Gulia, Shalaka Joshi, Palak Popat, Tanuja Shet

**Affiliations:** 1 Radiation Oncology, Homi Bhabha National Institute, Tata Memorial Centre, Dr Ernest Borges Rd, Parel East, Parel, Mumbai, Maharashtra 400012, India; 2 Statistics, Homi Bhabha National Institute, Tata Memorial Centre, Dr Ernest Borges Rd, Parel East, Parel, Mumbai, Maharashtra 400012, India; 3 Medical Oncology, Homi Bhabha National Institute, Tata Memorial Centre, Dr Ernest Borges Rd, Parel East, Parel, Mumbai, Maharashtra 400012, India; 4 Surgical Oncology, Homi Bhabha National Institute, Tata Memorial Centre, Dr Ernest Borges Rd, Parel East, Parel, Mumbai, Maharashtra 400012, India; 5 Radiology, Homi Bhabha National Institute, Tata Memorial Centre, Dr Ernest Borges Rd, Parel East, Parel, Mumbai, Maharashtra 400012, India; 6 Pathology, Homi Bhabha National Institute, Tata Memorial Centre, Dr Ernest Borges Rd, Parel East, Parel, Mumbai, Maharashtra 400012, India

**Keywords:** Onco-MPI, Geriatric-8, breast cancer, older patients, survival

## Abstract

**Purpose:**

Identifying vulnerabilities in older patients through comprehensive geriatric assessment is crucial but resource-intensive. This prospective study validated shorter tools- Onco-multidimensional prognostic index (MPI) and Geriatric-8 (G8) for predicting 2-year mortality in breast cancer patients and developed a nomogram using significant clinical predictors.

**Methods:**

The geriatric assessment was done in newly diagnosed treatment-naïve breast cancer patients with age ≥65 years. Overall survival was analysed by Kaplan-Meier test; univariate and multivariate analyses done to determine predictors of survival using Cox-proportional hazards model. A nomogram was constructed based on multivariate model and validated using calibration curve.

**Results:**

Among 300 patients (median age 70 years, IQR 67–74), 95.6% had ECOG-PS 0–1. High-grade histology and lympho-vascular invasion (LVI) positivity were seen in 77% and 30%, respectively. Most patients (77.6%) were non-metastatic. Luminal, HER2+ and triple negative subtype was present in 70%, 10.3% and 18.7% tumours. Rate of non-compliance to treatment was 25%. At median follow-up of 28.8 months, 2-year survival was 78%. The 2-year mortality for Onco-MPI categories was 13.36% (low-risk), 25.9% (medium-risk) and 29.8% (high-risk); high versus low-risk showed statistical difference (HR 2.68, 95% CI 1.13, 6.37; *p* = 0.026). G8 scores ≤14 and ≤12 both significantly predicted survival (*p* = 0.001; *p* = 0.005). On multivariate analysis, higher nodal stage, metastatic status, triple negative breast cancer subtype and LVI positivity independently predicted poorer survival. G8 score remained a significant predictor at both cut-offs (≤14 and ≤12) compared to Onco-MPI. A nomogram integrating nodal stage, metastatic status, LVI and G8 predicted 1- and 2-year survival with a C-index of 0.75 (95% CI: 0.65–0.84) and demonstrated good calibration at 12 and 24 months.

**Conclusion:**

In elderly breast cancer patients, G8 score was a stronger predictor of survival, outperforming onco-MPI. The nomogram combining conventional prognostic factors with G8 showed good discrimination for pre-treatment prognostication.

**Key summary points**

**Aim:** To evaluate the utility of Onco-MPI and G8 scores for predicting 2-year mortality in older breast cancer patients and to develop a nomogram using significant predictors for pre-treatment prognostication.

**Findings:** Onco-MPI and G8 identified high-risk patients but were not independent predictors of survival. A nomogram built on nodal stage, metastatic disease and presence of LVI with G8 tool (cut off 14) showed good discrimination and calibration.

**Message:** The developed nomogram with clinical predictors provides quick risk stratification and aids in survival prediction.

## Introduction

The United Nations (UN) predicts a two-fold increase in the number of old adults by 2050 and three-fold by 2100 due to increased life expectancy. In India, life expectancy increased from 62 to 72 years over the last 2 decades. Thereby, the incidence of cancer in older adults is also on the rise. The management of cancer in older adults is challenging due to the multidimensional deficits in physical and psychosocial function. A baseline geriatric assessment for identifying these deficits is strongly recommended for addressing them effectively. As a comprehensive geriatric assessment (CGA) is time and resource-intensive, various abbreviated tools like Multidimensional prognostic index (MPI), Geriatric-8 (G8) and onco-MPI have been developed and validated in diverse non-oncology and oncology populations. MPI covers functional, cognitive and nutritional status, as well as medical and social factors. The eight domains of MPI include activities of daily living (ADL), instrumental ADL (IADL), short portable mental status questionnaire, cumulative illness rating scale (CIRS)- comorbidity index (CIRS-CI), mini nutritional assessment, Exton Smith scale, number of medications and social support network. It has been validated for the prediction of mortality in non-oncological settings [1]. Another tool, onco-MPI, is a modification of MPI for oncological patients, developed and validated by Brunello *et al* [2]. It includes items like age, sex, ADL, instrumental IADL, Eastern cooperative oncology group (ECOG) performance status (ECOG-PS), mini-mental state examination (MMSE), body mass index (BMI), CIRS-CI, number of drugs, presence of the caregiver, cancer sites and cancer stage. Weights estimated from the Cox proportion model were assigned to each. The weighted sum ranged from 0 (low risk) to 1 (high risk). Onco-MPI has been validated in the Indian population for estimating short-term mortality in a retrospective analysis of a cancer cohort with different solid tumours [3]. Derived from the CGA tool, the G8 is a brief screening tool with eight items. It includes age, food intake, weight loss, mobility, neuropsychological problems, BMI, drugs and health status. For the validation of onco-MPI and G8 in the prediction of mortality, specifically in older breast cancer patients, this prospective study was conducted. In this cohort, we recently reported the results of validation of the various geriatric assessment tools for 1-year mortality [4]. Herein, we report the updated outcomes in the same cohort with mature follow-up. We also constructed a pre-treatment prognostic nomogram to estimate predicted survival.

## Materials and Methods

### Study population and methodology

This was a prospective observational study. Newly diagnosed breast cancer patients with age 65 years and older, who were treatment naïve, were accrued over 18 months from July 2021 to November 2022 at Tata Memorial Hospital, Mumbai. The study was approved by Institutional Ethics Committee (IEC-II) and registered with the Clinical Trials Registry-India (CTRI/2021/07/034792). The recruitment was done during the outpatient consultation and written informed consent was taken from all study subjects. The geriatric assessment using the Onco‑MPI and G8 tools was carried out by the study team member (doctor/research nurse) prior to any treatment. A full multidimensional comprehensive geriatric assessment was not performed. The demographic details, disease characteristics and treatment history were also recorded for every patient as available on the electronic medical records. The non-compliant or defaulted patients were defined as the patients who left the planned treatment incomplete or ones who did not start any form of treatment. The patients who were not considered fit for either of the treatment (surgery/chemotherapy/radiotherapy) by the oncologist even when indicated were not considered as non-compliant.

### Onco-MPI and G8 calculation

The onco-MPI score was calculated using the same weights as used by Brunello *et al* [2]. Using RECursive Partition and AMalgamation algorithm, the three predictive categories in their cancer cohort had a cut-off of 0–0.46 for Low risk, 0.47–0.63 for medium risk and 0.64–1 for high risk. The score for

G8 ranged between 0 and 17 with a score of <14 considered as vulnerable. Based on the validation study by Shah *et al* [5], cut-off score <12 was found to be appropriate in older Indian patients with cancer. Hence analysis was done for both the cut-offs.

### Statistical analysis

The demographic information, clinico-pathological parameters and treatment history was documented in proportions. Kaplan-Meier curves were used to examine the overall survival rate. The univariate analysis using the log-rank test was done for the conventional prognostic factors like molecular subtype of tumour, histology, grade, lympho-vascular invasion (LVI) status, tumour, node, metastasis staging (TNM) and treatment compliance status. Additionally, the individual geriatric domains of onco-MPI, including age, sex, BMI, ADL, IADL comorbidities, CIRS, MMSE, stage of disease, number of drugs used (all pre-treatment non-oncological medications), performance status and presence of caregiver along with onco-MPI score and G8 were studied. G8 is a screening tool and is composed of few questions assessing same domain (e.g., nutrition), hence univariate analysis was not done for individual questions. Multivariate analysis for risk estimation of significant factors was performed using the Cox proportional hazards model. The analysis was carried out with STATA software.

### Nomogram

A prognostic survival nomogram was constructed based on the final multivariate Cox model with pre-treatment clinical prognostic factors and geriatric tools to estimate 1- and 2-year survival probabilities. Each independent prognostic factor was assigned a weighted score proportional to its hazard ratio, and the total score corresponded to the predicted probability of survival at the specified time points. The model’s performance was evaluated using sensitivity, specificity, accuracy, the concordance index (C-index) and receiver operating curve (ROC) to assess discrimination, and calibration plots were generated by comparing predicted versus observed survival probabilities at 12 and 24 months.

## Results

A total of 300 patients were accrued. The median age of the study cohort was 70 years (IQR 67–74). Eighteen patients were ≥80 years, and maximum age in the cohort was 100 years. Of all the patients, 199 (66.3%) had known comorbidities. There were 287 (95.6%) patients with an ECOG performance status of 0–1. A total of 77% of the women had high-grade histology, 41.9% had LVI positivity and the majority (77.6%) had non-metastatic disease. Luminal tumours were seen in 210 (70%) patients, 31 (10.3%) were Her2 positive and 56 (18.7%) were with triple negative breast cancer (TNBC). The descriptive data are summarised in Table 1. A quarter 76 (25%) of the patients defaulted and/or did not comply with planned treatment, out of which 20 (26%) patients were metastatic.

Overall, 18 patients were not offered standard treatment. For surgery, only one patient was considered unfit. Eleven patients (3.6%) were considered unfit for chemotherapy (one for neoadjuvant and the rest for adjuvant). Radiotherapy was deferred in 6 (2%) patients. All the six patients in whom radiotherapy was deferred were post mastectomy with favourable histopathological features like single node positivity with T1/2 tumours and strong hormone positivity. All of them had multiple comorbidities, except one all were >70 years while poor wound healing was present in two of them. For the onco-MPI, 44 (15%), 135 (45%) and 121 (40%) cases were low, medium and high risk, respectively. For the G8, 112 (37.3%) patients were under high risk with score ≤14. Considering the cut off ≤12, 46 (15.3%) were under high risk.

**Figure 1.**
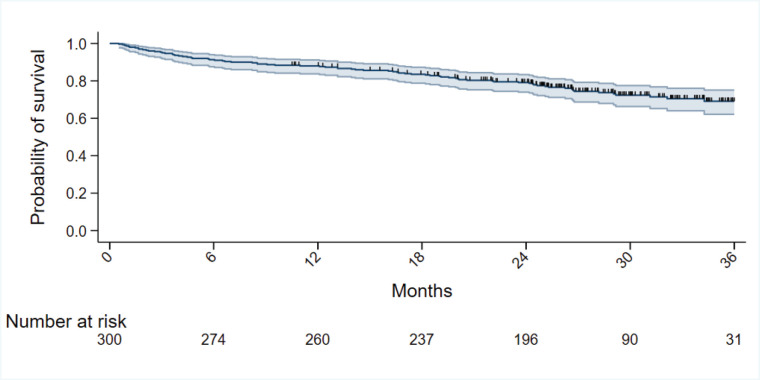
Kaplan Meier curve for overall survival.

**Figure 2.**
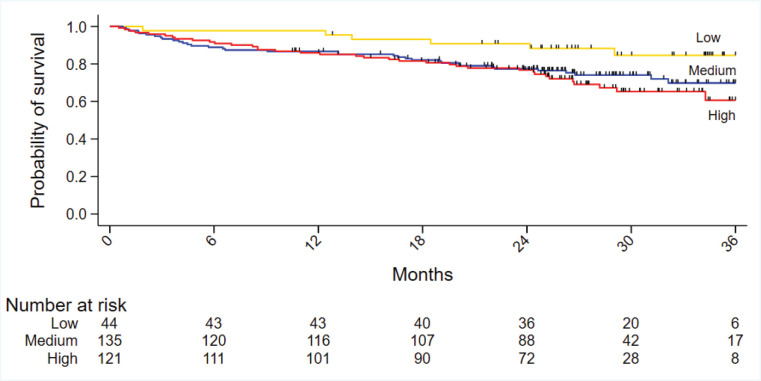
Kaplan Meier curves for 2-year mortality with Onco-MPI.

**Figure 3.**
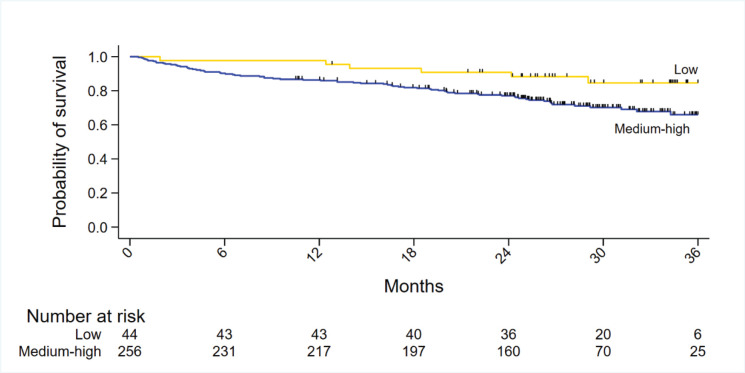
Kaplan Meier curve for 2-year mortality merged Onco-MPI category.

The median follow-up was 28.8 months (95% CI 27.3, 29.8). The 1-year mortality data has been published previously, which showed that among the three tools, only G8 was predictive of 1-year mortality in both univariate and multivariate analysis [4]. Currently, we analysed 2-year mortality as the cohort has a mature follow up. The 2-year overall survival was 78% (Figure 1). Across the onco-MPI categories, the 2-year mortality was 13.36% (low risk), 25.9% (medium risk) and 29.8% (high risk). The patients in medium risk were at higher risk of mortality compared to patients in low risk category (HR 2.21, 95% CI 0.93, 5.25), however, not statistically significant (*p* = 0.07). A significant survival difference was seen between low versus high risk category (HR 2.68, 95% CI 1.13, 6.37; *p* = 0.026) (Figure 2). When the medium and high-risk categories of onco-MPI were merged, the difference in survival (HR 2.42, 95% CI 1.05, 5.58; *p* = 0.038) compared to the low-risk category was significant (Figure 3).

**Figure 4.**
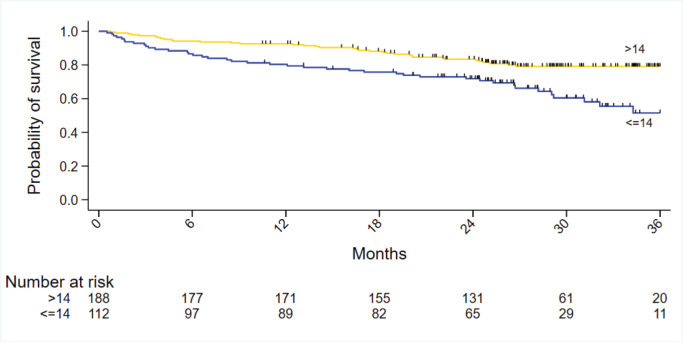
Kaplan Meier curves for 2-year mortality for G8 with cut off ≤14.

**Supplement Figure 1.**
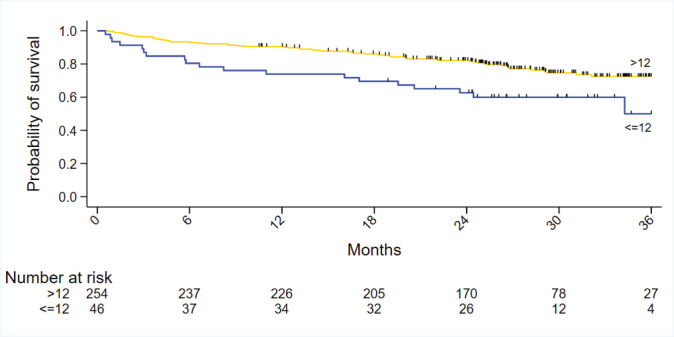
Kaplan Meier curves for 2-year mortality for G8 with cut off ≤12.

The univariate analysis for the individual domains in onco-MPI showed significant mortality correlation only for age, ADL, IADL and MMSE amongst all the domains. There was no significant difference seen for comorbidities, stage, CIRS, ECOG status, BMI and number of medications (Table 2). For the G8 scores, both cut-off of ≤14 and ≤12 predicted the 2-year survival (*p* = 0.001 and *p* = 0.005, respectively) (Figure 4; Supplement Figure 1). Among the conventional prognostic factors, on univariate analysis, survival was predicted by age, TNM staging, grade of tumour, molecular subtype, LVI status and treatment compliance (Table 3).

Multivariate analysis was done for the conventional prognostic factors and for the geriatric tools. For conventional factors, it was observed that higher nodal stage, metastatic disease status, TNBC molecular subtype and LVI positivity significantly predicted poor survival (Table 4). For the individual domains of onco-MPI, it was seen that only higher age and stage were associated with poor survival (Supplement Table 1). On multivariate analysis for the geriatric tools, G8 score with cut off of 14 and 12 showed statistically significant results when individually compared to onco-MPI (low vs medium and high risk) (Table 5).

**Figure 5.**
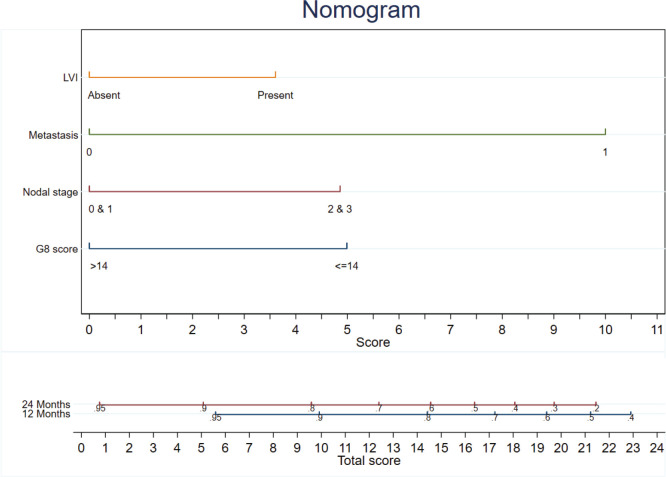
Nomogram based on multivariate Cox proportional hazards model.

**Figure 6.**
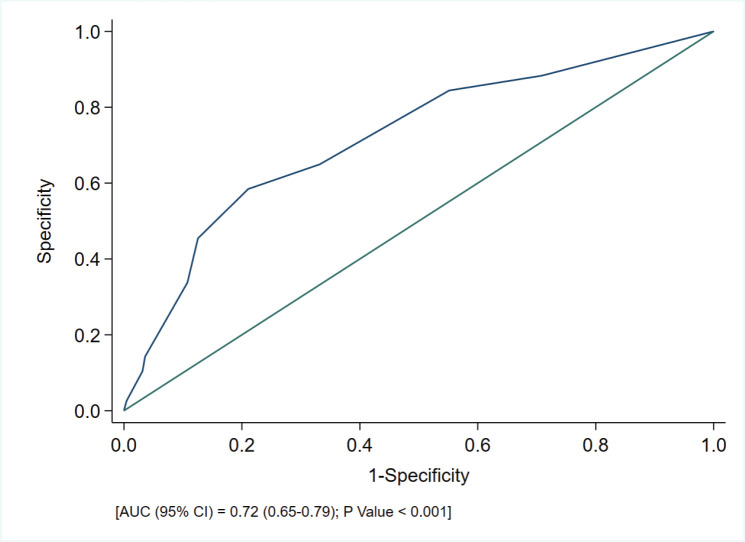
ROC for the nomogram.

**Figure 7.**
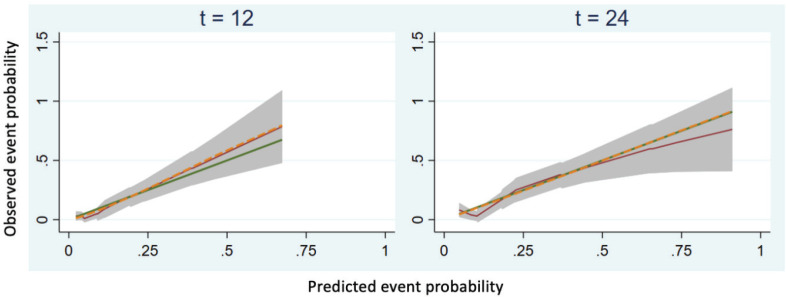
Calibration plot for 1- and 2-year time point.

A nomogram was built using the multivariate model combining the conventional prognostic factors, including nodal stage, metastatic disease and presence of LVI with G8 tool (cut off 14) to estimate 1 and 2-year survival probabilities, with a concordance index (C-index) of 0.75, 95% CI 0.65, 0.84 (Figure 5). Each variable was assigned a score based on its hazards ratio. The score ranged from 0 to 24, with a higher score indicting poorer survival. Using the ROC curve (Figure 6) and Youden’s index, cut-off of score ≥10 was found to be an optimal cut-off. It showed moderate sensitivity (58%) and good specificity (79%), with accuracy of 74%. The calibration plot (Figure 7) shows that at 1 and 2 years, the predictive probability curve (dashed line) closely follows the line of calibration with shaded area representing the 95% CI for observed probabilities indicating that the model demonstrates good calibration.

## Discussion

Geriatric assessment is a domain-oriented assessment. Comprehensive Geriatric Assessment is very time-consuming and difficult to implement routinely in busy clinics. Though shorter tools like G8, VES, MPI and onco-MPI have been developed and validated in different non-oncological and oncological populations to identify vulnerabilities, predict mortality and tailor treatment, it is important to validate in the cancer population of interest before it can be used to guide clinical decisions [1, 2, 3, 5]. Our study showed a significant correlation of onco-MPI categories with mortality but only when the medium and high-risk categories were combined (HR 2.42, 95% CI 1.05, 5.58; *p* = 0.038) (Figure 3). The BMI, ECOG-PS and MMSE domains under onco-MPI do not show a correlation with mortality, probably due to the key differences in the demographic factors of the cancer population studied by Brunello *et al* [2] and the breast cancer cohort in our study. Brunello *et al* [2] developed and validated onco-MPI in a cancer cohort comprising of different cancer sites (it also included 46% breast cases). Age, male sex, poorer MMSE, worse ADL and IADL, the number of severe comorbidities, poor ECOG PS, lack of a caregiver and late-stage cancer were all found to be associated with greater 1-year mortality. Even in their study, a lower mortality was observed for breast cancer diagnosis (vs other cancer sites) and higher BMI (which is contradictory).

Only one study has been done to validate the predictability of onco-MPI in Indian cohort, by Shenoy *et al* [3], which was from Geriatric clinic of our institution. The 1-year mortality rates for low-risk patients were significantly different from those for medium- and high-risk patients, according to this retrospective study (40.6% versus 53.1% versus 71.7%; *p* < 0.001). It included patients with any solid tumour who underwent geriatric assessment, of age 60 years and above. Majority of patients had lung and esophageal primary (~63%). The breast cancer patients comprised only 1% of the population. Moreover, 75% were males (versus 1.3% in this study) , 41.4% cases having ECOG 2 or 3 (versus 4.4% in this study) and 54.4% with metastatic disease (versus 22.4% in this study). Our cohort had 95.6% of patients with ECOG PS of 0–1 which correlates with the study published by Kumar *et al* [6] reporting the ECOG PS in patients diagnosed with different solid tumours, aged 18–101 years (Median age 66), in which 94% of breast cancer patients had PS of 0–1. This suggests that breast cancer patients are much fitter compared to patients diagnosed with other primaries.

The recent data of older patients with advanced urothelial cancer assessed by onco-MPI was published as an abstract [7]. Only 4% of patients were under high-risk category of onco-MPI, hence, they concluded that there is a need to set new cut-offs for specific populations. Similarly, our study was carried out in only breast cancer patients, but 40% cases were in the high-risk category as compared to 4% reported by Bimbatti *et al* [7]. Significant correlation of onco-MPI with 2-year mortality was observed but only when medium and high-risk categories (score 0.47–1) were combined. This means that cut-offs can be varied to derive stronger statistically significant results, implying that it might be more effective to stratify patients into two risk categories rather than three. Furthermore, onco-MPI has been developed and validated in a much older population (median 77 years) with different solid tumours for 1-year mortality. On the contrary, it is not seen to be predictive of either 1-year [4] or 2-year mortality in younger (median age 70 years) and relatively fitter (95% having ECOG 0–1) geriatric breast cancer patients.

G8 is a short screening tool designed to identify vulnerable patients who would benefit from complete geriatric assessment [8]. This study included patients aged 70 years or older with various malignancies who received first line chemotherapy; however, patients with breast cancer were excluded as very few had received first line chemotherapy. A strong sensitivity estimate (85%) was obtained with the G8 tool's cut off value of ≤14, with a specificity of 65%. A study done in multi-ethnic Asian population with age 65 years or more, showed G8 performed well in identifying older adults with cancer who would benefit from a CGA [9]. An observational retrospective study in Indian cohort of age 60 years and older patients [5], showed that cut-off of score ≤14 did not predict survival outcomes (*p* = 0.28), however on lowering the cut off to ≤12 had significant association with survival (*p* = 0.004). The lower cut off allowed 35% reduction in the number of patients undergoing a complete geriatric assessment. In both of these studies, breast cancer patients were under represented. In our cohort, comprehensive geriatric assessment was not done in these patients and hence sensitivity and specificity could not be determined. However, both cut-offs of score 14 and 12 showed significant prediction of survival, outperforming onco-MPI. Hence, this can be a starting point for geriatric assessment in busy oncology clinics where services need to be triaged.

The nomogram built with nodal stage, metastatic disease and LVI with G8 score with cut-off of 14 combines the oncological and geriatric parameters providing holistic and clinical risk stratification. This may aid in personalising treatment intensity, counselling patients and identifying those who may benefit from geriatric intervention or closer follow-up. A retrospective study by Kanesvaran *et al* [10] in patients with cancer aged 70 years and older, age, albumin, ECOG, geriatric depression scale, stage and DETERMINE nutritional index were independent predictor of survival. A nomogram was derived from these which has a concordance index of 0.71. This was also externally validated in 249 patients later [11]. The key novelty of our nomogram is that it is integrating the geriatric tool with the conventional tumour-related prognostic factors. Conventional breast cancer prognostication does not capture frailty and physiological reserve, while G8 identifies vulnerability without reflecting tumour aggressiveness. Combining them provides more comprehensive, complete and clinically meaningful risk assessment rather than using either alone.

There are certain limitations in this study. The study was conducted in a single tertiary cancer centre, which may limit generalisability. CGA was not performed, which is the gold standard, preventing comparison of the G8 and onco-MPI against full CGA. The nomogram showed a moderate sensitivity and requires external validation before clinical implementation.

## Conclusion

In this exclusive cohort of breast cancer who were more fitter and younger, both onco-MPI and G8 identified high-risk patients; however, G8 score was a stronger predictor of survival outperforming onco-MPI. The nomogram combining the G8, with conventional prognostic factors including nodal stage, metastatic disease and LVI showed good discrimination and calibration, enabling quicker pre-treatment risk stratification for predicting survival.

## Conflicts of interest

None.

## Funding

None.

## Ethical approval

The study was approved by the Institutional Ethics Committee (IEC-II) and registered with the Clinical Trials Registry-India (CTRI/2021/07/034792).

## Figures and Tables

**Table 1.** Descriptive data of cohort.

Variable	Category	Value
Age (years)	Median (IQR)	70 (67–74)
Sex	Female	296 (98.7%)
	Male	4 (1.3%)
Comorbidities	No	101 (33.7%)
	Yes	199 (66.3%)
Tumor stage (T stage)	1	13 (4.4%)
	2	117 (39%)
	3	58 (19.3%)
	4	112 (37.3%)
Nodal stage (N stage)	0	80 (26.7%)
	1	122 (40.6%)
	2	53 (17.7%)
	3	45 (15%)
Metastatic stage (M stage)	0	233 (77.6%)
	1	67 (22.4%)
Grade of tumor	2	55 (18.3%)
	3	231 (77%)
	NA	14 (4.7%)
ECOG PS	0	199 (66.3%)
	1	88 (29.3%)
	2	11 (3.7%)
	3	2 (0.7%)
LVSI status	Present	90 (30%)
Treatment completion	Yes	224 (74.7%)
	No	76 (25.3%)
Molecular subtypes	Luminal A	158 (52.7%)
	Luminal B	43 (14.3%)
	HER 2	31 (10.3%)
	TNBC	56 (18.7%)
	NA	12 (4%)
Surgery	Mastectomy	168 (56%)
	Breast conservative surgery	48 (16%)
	Non-compliant	19 (6.3%)
	Not indicated	57 (19%)
	Death before surgery	8 (2.7%)
Chemotherapy	￼Neoadjuvant	38 (12.7%)
	Adjuvant	77 (25.7%)
	Neoadjuvant + Adjuvant	30 (10%)
	Palliative	25 (8.3%)
	Non-compliant	32 (10.7%)
	Not indicated	94 (31.3%)
	Death before chemotherapy	4 (1.3%)
Radiotherapy	Adjuvant	135 (45%)
	Palliative	11 (3.7%)
	Non-compliant	41 (13.7%)
	Not indicated	96 (32%)
	Death before radiotherapy	17 (5.7%)
Onco-MPI	Low risk	44 (14.7%)
	Medium risk	135 (45%)
	High risk	121 (40.3%)
G8	>14	188 (62.7%)
	≤14	112 (37.3%)
	>12	254 (84.7%)
	≤12	46 (15.3%)

**Table 2.** Univariate analysis for individual domains of Onco-MPI.

Variable	Category	*N*	Event (*N*)	HR (95% CI)	*p*-value
Age	Cont variable	300	77	1.05 (1.01, 1.09)	0.010*
Sex	Female	296	76	—	
	Male	4	1	0.78 (0.11, 5.61)	0.805
BMI	Cont variable	300	77	0.97 (0.92, 1.01)	0.139
ADL	Cont variable	300	77	0.71 (0.61, 0.84)	**<0.001**
IADL	Cont variable	300	77	0.87 (0.79, 0.94)	**0.001**
Comorbidities	No	101	32	—	
	Yes	199	45	0.66 (0.42, 1.04)	0.076
CIRS	Cont variable	300	77	0.95 (0.85, 1.07)	0.379
MMSE	Cont variable	300	77	0.96 (0.93, 1.00)	**0.030**
Stage	I-III	232	54	—	
	IV	67	23	1.62 (0.99, 2.64)	0.053
Number of drugs used	Cont variable	300	77	0.90 (0.78, 1.03)	0.112
ECOG PS	0	199	52	—	
	1, 2 & 3	101	25	0.97 (0.60, 1.57)	0.909
Presence of caregiver	yes	231	64	—	
	no	69	13	0.66 (0.36, 1.19)	0.168

* bold indicates the values that are significant

**Table 3.** Univariate analysis for onco-MPI and Geriatric-8, and conventional prognostic factors.

Variable	Category	*N*	Event (*N*)	HR (95% CI)	*p*-value
Onco-MPI	Low risk	44	6	—	
	Moderate risk	135	35	2.21 (0.93, 5.25)	0.073
	High risk	121	36	2.68 (1.13, 6.37)	**0.026**
Onco-MPI	Low risk	44	6	—	
	Moderate/high risk	256	71	2.42 (1.05, 5.58)	**0.038**
G8	>14	188	36	—	
	≤14	112	41	2.17 (1.39, 3.40)	**0.001**
G8	>12	254	58	—	
	≤12	46	19	2.10 (1.25, 3.53)	**0.005**
T stage	0, 1 & 2	130	22	—	
	3 & 4	170	55	2.17 (1.32, 3.56)	**0.002**
N stage	0 & 1	202	36	—	
	2 & 3	98	41	2.69 (1.72, 4.22)	**<0.001**
M stage	0	233	46	—	
	1	67	31	3.12 (1.97, 4.94)	**<0.001**
Grade	2	55	5	—	
	3	231	66	3.40 (1.37, 8.45)	**0.008**
Molecular subtype	HR positive/Her2Nu positive	244	51	—	
	TNBC	56	26	2.51 (1.56, 4.02)	**<0.001**
LVI status	Absent	125	17	—	
	Present	90	22	1.94 (1.03, 3.65)	**0.040**
Treatment status	Yes	224	38	—	
	No	76	39	5.19 (3.29, 8.16)	**<0.001**

**Table 4.** Multivariate cox regression for conventional factors.

Variable	*N*	Event N	HR^1^	95% CI^1^	*p*-value
Nodal stage					
N0-1	201	36	—	—	
N2-3	98	41	2.21	1.14, 4.28	**0.019**
Metastatic stage					
0	233	46	—	—	
1	67	31	4.76	2.27, 9.98	**<0.001**
Molecular subtype					
Others	244	51	—	—	
TNBC	56	26	2.06	1.04, 4.11	**0.039**
LVI					
Negative	125	17	—	—	
Positive	90	22	2.15	1.12, 4.13	**0.022**

1 HR = Hazard Ratio, CI = Confidence Interval

**Table 5.** Multivariate Cox regression for Onco-MPI and G8 cut off 14.

Variable	*N*	Event N	HR^1^	95% CI^1^	*p*-value
Onco-MPI					
Low	44	6	—	—	
Moderate-high	256	71	2.02	0.87, 4.70	0.103
G8					
>14	188	36	—	—	
<=14	112	41	2.01	1.28, 3.17	**0.003**

1 HR = Hazard Ratio, CI = Confidence Interval

**Supplement Table 1.** Multivariate Cox regression for Onco-MPI domains.

Variable	*N*	Event N	HR^1^	95% CI^1^	*p*-value
Age					
Continuous variable	300	77	1.04	1.00, 1.08	**0.036**
MMSE					
Continuous variable	300	77	0.97	0.94, 1.01	0.160
Stage					
I–III	232	54	—	—	
IV	67	23	1.72	1.05, 2.82	**0.030**
Comorbidities					
No	125	17	—	—	
Yes	90	22	2.15	1.12, 4.13	0.135

1 HR = Hazard Ratio, CI = Confidence Interval
